# Budapest and Buda-Pest

**Published:** 1909-09-25

**Authors:** W. H. Shrubsole

**Affiliations:** 22 Halons Road, Eltham


### Editor's Letter-Box.

### BUDAPEST AND BUDA-PEST.

Dear Sir,—As a friend of the Hungarian people, I am much gratified by your sympathetic article on the Medical Congress at Budapest. In reading it over I notice that you have used the obsolete form of the name of the capital city. Bud-Pest implies two cities, whereas there is now but one city, under the control of one authority. For more than thirty years Budapest has been the name of the whole of the capital, and it applies to every part of it on both banks. As the name was given and is used by Hungarians, I submit that it should be adopted and used by other nations.—Yours very truly,

W. H. Shrubsole.

22 Halons Road, Eltham, September 13, 1909.

